# HIV-1 and HIV-2 prevalence, risk factors and birth outcomes among pregnant women in Bissau, Guinea-Bissau: a retrospective cross-sectional hospital study

**DOI:** 10.1038/s41598-020-68806-5

**Published:** 2020-07-22

**Authors:** Dlama Nggida Rasmussen, Noel Vieira, Bo Langhoff Hønge, David da Silva Té, Sanne Jespersen, Morten Bjerregaard-Andersen, Inés Oliveira, Alcino Furtado, Magarida Alfredo Gomes, Morten Sodemann, Christian Wejse, Holger Werner Unger

**Affiliations:** 1 Department of Public Health, Research Unit of General Practice, University of Southern Denmark, Winsløwparken 19.2, 5000 Odense, Denmark; 2 Department of Infectious Diseases, Odense University Hospital, Odense, Denmark; 3 Bandim Health Project, INDEPTH Network, Bissau, Guinea-Bissau; 4 Association Ceu e Terras, Bissau, Guinea-Bissau; 5 Department of Clinical Immunology, Aarhus University Hospital, Aarhus, Denmark; 6 Department of Infectious Diseases, Aarhus University Hospital, Aarhus, Denmark; 7 National HIV Programme, Secretariado Nacional de Luta Contra le Sida, Ministry of Health, Bissau, Guinea-Bissau; 8 Department of Endocrinology, Hospital of South West Denmark, Esbjerg, Denmark; 9 Research Center for Vitamins and Vaccines, Statens Serum Institut, Copenhagen, Denmark; 10 Department of Obstetrics and Gynaecology, Simão Mendes National Hospital, Bissau, Guinea-Bissau; 11 GloHAU, Center for Global Health, Department of Public Health, Aarhus University, Aarhus, Denmark; 12 Centre for Maternal and Newborn Health, Liverpool School of Tropical Medicine, Liverpool, United Kingdom; 13 Menzies School of Health Research, Darwin, Australia; 14 Department of Obstetrics and Gynaecology, Royal Darwin Hospital, Darwin, Australia

**Keywords:** HIV infections, Epidemiology

## Abstract

The human immunodeficiency virus (HIV) remains a leading cause of maternal morbidity and mortality in Sub-Saharan Africa. Prevention of mother-to-child transmission (PMTCT) has proven an effective strategy to end paediatric infections and ensure HIV-infected mothers access treatment. Based on cross-sectional data collected from June 2008 to May 2013, we assessed changes in HIV prevalence, risk factors for HIV, provision of PMTCT antiretroviral treatment (ART), and the association between HIV infection, birth outcomes and maternal characteristics at the Simão Mendes National Hospital, Guinea-Bissau’s largest maternity ward. Among 24,107 women, the HIV prevalence was 3.3% for HIV-1, 0.8% for HIV-2 and 0.9% for HIV-1/2. A significant decline in HIV-1, HIV-2, and HIV-1/2 prevalence was observed over time. HIV infection was associated with age and ethnicity. A total of 85% of HIV-infected women received ART as part of PMTCT, yet overall treatment coverage during labour and delivery declined significantly for both mothers and infants. Twenty-two percent of infants did not receive treatment, and 67% of HIV-2-infected mothers and 77% of their infants received ineffective non-nucleoside reverse transcriptase inhibitors for PMTCT. Maternal HIV was associated with low birth weight but not stillbirth. Inadequate continuity of care and ART coverage present challenges to optimal PMTCT in Guinea-Bissau.

## Introduction

Despite remarkable progress over the past decade, Sub-Saharan Africa (SSA) continues to have the highest global prevalence of HIV. Women remain disproportionately at risk of acquiring HIV, with an estimated 59% of all new infections occurring amongst females^1^. Furthermore, HIV remains one of the leading causes of mortality among women of reproductive age in the region^2^. In 2017, an estimated 1.3 million pregnant women were living with HIV, and 130,000 infants were infected with HIV in SSA^3^.

Approximately 90% of all new paediatric infections occur during the perinatal period, of which 50% occur in relation to labour and delivery^4,5^. Antiretroviral treatment (ART) as part of prevention of mother-to-child transmission (PMTCT) is a key strategy to combat the African HIV epidemic and decreases vertical transmission rates from as high as 45% to < 5% in breastfeeding populations^6^. PMTCT has yielded remarkable results, with an estimated 93% of pregnant women in eastern and southern Africa living with HIV receiving antiretroviral prophylaxis, resulting in mother-to-child transmission rates of less than 10%. Nevertheless, western and central Africa appear to lag behind, with fewer than half (48%) of pregnant women living with HIV in 2017 receiving PMTCT services^1^.

Guinea-Bissau is a West African country populated with 1.8 million inhabitants, of which 400,000 reside in the capital Bissau. According to the joint United Nations programme on HIV/AIDS (UNAIDS), 3.4% (95% CI 2.6–3.8) of the population in Guinea-Bissau is infected with HIV (UNAIDS, 2018c), yet the true number may be notably higher considering that HIV prevalence in the country’s capital is 6.7%^7^. A previous study among pregnant women conducted from 2002 to 2006 found that 5.7% were HIV-1 infected, and 2.4% were HIV-2 infected, including 0.7% HIV-1/2 dually infected women^8^.

The Ministry of Health in Guinea-Bissau implemented a national HIV program in 2005, providing free of charge ART. Yet, more than a decade later, the country remains far from achieving the 90–90–90 targets^1^. The delivery of HIV treatment has been impeded by numerous barriers including political instability, frequent HIV clinic relocations and inadequate drug supply resulting in treatment interruptions^9^. In addition, poor laboratory capacity, inadequate validation of HIV rapid tests, repeated unavailability of CD4 cell count measurements, and lack of access to HIV-RNA measurement have delayed initiation of ART and led to the identification of treatment failure^9,10^. As a result, UNAIDS estimated that only 32% of adults in the country accessed antiretroviral therapy in 2018^11^.

PMTCT counselling and testing has been available at selected antenatal clinics (ANC) in Bissau since 2002^8^. Between October 2007 and April 2013, 86% of pregnant women had at least one antenatal visit, but only 54% attended all four recommended visits in Bissau. In addition, only 29% of women in rural areas and 69% of women in urban settings delivered at health facilities in the country^12^. Previous qualitative research from Guinea-Bissau has shown that a lack of HIV and PMTCT knowledge, customary and cultural beliefs associated with HIV and ill health, HIV stigma and discrimination, and fear of partnership dissolution were common barriers to PMTCT^13^. In addition, a study exploring peripartum opt-out testing found that regular stock interruptions and political instability negatively affected the provision of PMTCT^14^. High rates of early mortality and loss to follow-up have recently been described among HIV-infected children (< 15 years of age) in Guinea-Bissau. Based on a study of 525 HIV-infected children, 11% died and 39% were lost to follow-up within the first year of follow-up, highlighting continuous challenges with implementing sustainable PMTCT services^15^. Consequently, there remains a paucity of research exploring the delivery of PMTCT services with the overall aim of improving treatment and care for HIV-infected mothers and their children in Guinea Bissau.

In this retrospective study, we assessed changes in HIV-1 and HIV-2 prevalence, factors associated with HIV infection, birth outcomes and the provision of ART as part of PMTCT services over a 5-year period at the national maternity ward in Bissau, Guinea-Bissau.

## Methods

### Study setting

The study was conducted by the Bandim Health Project (BHP) (https://www.bandim.org) at the Simão Mendes National Hospital (HNSM) maternity ward located in Bissau. The BHP, a health and demographic surveillance site present in Guinea-Bissau for 4 decades, routinely collects demographic and clinical data on all deliveries at HSNM. This public facility is the principal provider of comprehensive obstetric care in Guinea-Bissau, and approximately 90% of women who deliver at the facility are residents of the country’s capital, Bissau.

From June 2008 onwards, opt-out HIV counselling and testing has been offered to women admitted to the maternity ward for the management of labour- and delivery- and/or pregnancy-related complications when HIV tests were available. HIV testing and ART were free of charge and provided by the Guinean Ministry of Health, but all women paid a flat fee of 2000 XOF (3–4 USD) to give birth at HNSM^14^. All newly diagnosed women and their infants were referred to the Italian-Guinean NGO-clinic, Ceu e Terras, or to other local HIV centres for follow-up, counselling, and further PMTCT ART^13^. Data registration of testing for HIV at delivery ceased in May 2013 due to funding constraints. While HIV testing at labour and delivery was national policy at the time of the study, not all women were tested. We have previously described the characteristics of women tested compared with those not tested ^14^.

### Study design and participants

We conducted a retrospective cross-sectional survey exploring HIV prevalence, risk factors for HIV, treatment provision, and birth outcomes (low birth weight, LBW, and stillbirth), drawing on data routinely collected through the BHP surveillance system at HNSM from June 2008 until May 2013. All women presenting to HNSM for delivery or immediate postpartum care who had been tested for HIV were included in this study.

### Data collection

Data were recorded in the HNSM maternity ward registration system and included basic socio-demographic and clinical data. Data cleaning was performed daily (including weekends) by trained research assistants from BHP who also collected supplementary demographic and clinical information using separate case report forms (CRFs). We have previously described the data collected in detail^14,16^. During the study period, it was national policy that hospital midwives offer immediate HIV counselling and testing to all women presenting to the maternity for delivery or, as many women had not attended ANC or did not receive PMTCT testing or counselling during pregnancy. Midwives had been trained to complete a short CRF as part of the counselling and testing routine to collect data on previous HIV testing, known sero-status, and ART use^14^.

### HIV testing

HIV screening was performed using the Determine® HIV-1/2 rapid test (Abbot Diagnostics, Maidenhead, United Kingdom). To confirm infection and to discriminate between HIV types, women with positive and inconclusive screening results were subsequently tested with another rapid test, SD Bioline HIV-1/2 3.0 (Standard Diagnostics, Kyonggi-do, South Korea).

### HIV treatment regimens

Provision of PMTCT at the initiation of this survey was guided by the 2006 World Health Organization (WHO) recommendations, i.e., ART prophylaxis in the third trimester (28 weeks) of pregnancy consisting of a regimen of twice daily Zidovudine (AZT), single-dose nevirapine (sd NVP) at onset of labour and Zidovudine plus Lamivudine (AZT + 3TC) for 1 week from birth. Women diagnosed in labour were given a single-dose nevirapine followed by AZT + 3TC for 1 week starting in labour. For women who needed treatment for their own health, triple ART (cART) was initiated as soon as possible. For HIV-2 and HIV-1/2 dually infected mothers, a combination of (AZT + 3TC) + Lopinavir/Ritonavir was recommended. For infants, guidelines recommended sd NVP plus AZT twice a day for either 4 weeks if the mother had received less than 4 weeks AZT prophylaxis prior to labour or for 1 week if the mother had received at least 4 weeks AZT prophylaxis. Infants of mothers receiving cART were recommended AZT twice daily^17^. After 2010, in line with the revised WHO recommendations, all pregnant women with CD4 count levels < 350 cells/mm^3^, irrespective of clinical stage, and women with a WHO clinical stage 3 or 4 infection, were started on triple ART. In addition, women not receiving ART for their own health, received (Option A) prophylaxis with AZT as monotherapy from as early as 14 weeks of pregnancy plus a single dose NVP + AZT + 3TC during labour and delivery followed by AZT + 3TC for 7 days after delivery, or triple ART prophylaxis (Option B) from as early as 14 weeks of pregnancy. The infant regime consisted of daily NVP from birth for a minimum of 4–6 weeks, and until 1 week after all exposure to breastmilk had ended. For infants receiving replacement feeding only daily NVP or sd NVP + daily AZT from birth and until 4–6 weeks of age was recommended^18^. Due to logistical issues, treatment was often started without CD4 cell count measurements based on clinical assessment. Throughout the study period, repeated stock outages and lack of ART at times forced clinicians to switch treatment and often to provide more simple regimes than recommended^9, 19^. In 2015, Guinea-Bissau began implementing option B+, i.e., lifelong ART from diagnosis^20,21^.

### Statistical methods

Maternity and HIV testing data were entered in password-secured databases (dBase 5.0, dataBased Inc, Vestal, NY, USA; Microsoft Access 2007, Microsoft, Redmond, WA, USA), and datasets were merged using unique birth numbers. The data were analysed using Stata 14.0 (Stata Corporation, College Station, TX, USA). Outcome variables such as LBW and stillbirths were dichotomized (0 = absent, 1 = present). Continuous explanatory variables were grouped categorically. Factors associated with HIV serostatus and birth outcomes were determined using univariate and multivariate logistical regression models. The multivariate analysis was fitted with statistically significant covariates (as determined by Wald’s test). Birth outcomes i.e., stillbirths and low birthweight (LBW) were examined according to HIV status and adjusted for significant covariates. In accordance with the WHO criteria, stillbirth was defined as a newborn at or above 1,000 g showing no vital signs immediately after birth (Apgar score = 0)^22^. Low birth weight was defined as birth weight of < 2,500 g^23^. Miscarriages (birth weight < 1,000 g) were excluded. In the birth outcome analysis, “births” pertain to the number of fetuses at risk. Therefore, the number of total births is higher than the number of pregnant women due to multiple pregnancies. In the logistic regression models, adjustment for birth outcomes was made for clustering of twins using a specific pair number. Inconclusive test results were categorized as HIV-negative. Missing values were included in the logistic regression models. Trends over time (calendar year) for HIV prevalence and treatment were determined using Pearson’s χ^2^ test. Due to a lack of data to confirm self-reported antenatal treatment regimens and reasons for selecting a given treatment at labour, we opted to assess antenatal and treatment at labour separately. A *p* value of < 0.05 was considered significant.

### Ethical considerations

All patient data were fully anonymised before being accessed. The use of government surveillance maternity data was approved by the National Ethical Committee in Guinea-Bissau (CNES-2010-018). A separate ethical approval was obtained for the analysis of HIV data and linkage of delivery and HIV databases (CNES-2011-030). The study was carried out in accordance with Guinea-Bissau Medical Research Ethics Committee requirements and the Helsinki declaration. All participants were counselled and asked for verbal informed consent before data collection and HIV testing. Newly diagnosed women were offered ART at labour and subsequently referred for follow-up. The manuscript was prepared in accordance with the STROBE guidelines (Supplemental Checklist 1).

## Results

### Participant characteristics

**Figure 1:**
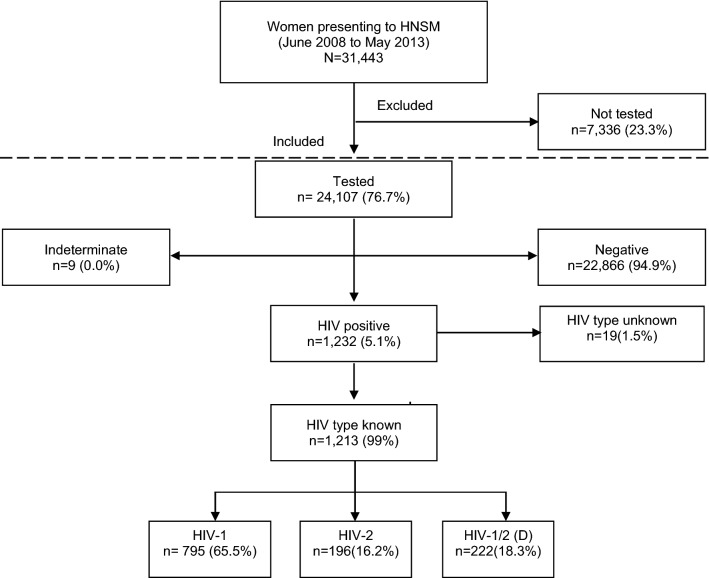
Flow diagram of participants included in the study. The flow diagram displays women included in this study according to HIV status.

From June 2008 to May 2013, a total of 31,443 women presented to the HNSM maternity ward for delivery and/or pregnancy-related care, of which 77% (n = 24,107) were tested for HIV (Fig. 1). The median age of women was 24 years (inter-quartile range [IQR] 20–29, range 13–49). The principal ethnic groups were the Balanta (23%) and the Fula (22%). Forty-five percent of women had no or little (primary) education, and 19% reported a polygamous marriage (Table 1).

**Table 1:** Baseline characteristics of pregnant women tested for HIV stratified by HIV-1, HIV-2 and HIV-1/2 status from June 2008 to May 2013 (N = 24,107).

Baseline characteristics	Study population	HIV-1 positive		HIV-2 positive		HIV-1/2 positive	
	N = 24,107 (col%)	n = 795 (row%)	*p*	n = 196 (row%)	*p*	n = 222 (row%)	*p*
**Age**			**< 0.001**		**< 0.001**		**< 0.001**
Age 13–19	5,655 (23.5)	68 (1.2)		11 (0.2)		11 (0.2)	
Age 20–24	7,215 (29.9)	234 (3.2)		38 (0.5)		55 (0.8)	
Age 25–29	6,238 (25.9)	270 (4.3)		63 (1.0)		80 (1.3)	
Age 30+	4,999 (20.7)	223 (4.5)		84 (1.7)		76 (1.5)	
**Ethnic group**			**< 0.001**		0.749		**0.016**
Balanta	5,476 (22.7)	189 (3.5)		42 (0.8)		37 (0.7)	
Bijagos	436 (1.8)	9 (2.1)		3 (0.7)		4 (0.9)	
Felupe	374 (1.6)	7 (1.9)		3 (0.8)		1 (0.3)	
Fula	5,262 (21.8)	159 (3.0)		37 (0.7)		46 (0.9)	
Mancanha	1,878 (7.8)	62 (3.3)		15 (0.8)		20 (1.1)	
Mandinga	2,485 (10.3)	85 (3.4)		27 (1.1)		39 (1.6)	
Manjaco	1,860 (7.7)	55 (3.0)		17 (0.9)		19 (1.0)	
Mixed ethnicity	682 (2.8)	23 (3.4)		9 (1.3)		9 (1.3)	
Pepel	3,495 (14.5)	91 (2.6)		29 (0.8)		26 (0.7)	
Saracule	252 (1.1)	6 (2.4)		3 (1.2)		–	
Other^**a**^	1,886 (7.8)	107 (5.7)		11 (0.6)		21 (1.1)	
NA	21 (0.1)	2 (9.5)		0 (0.0)		–	
**Marital status**			**< 0.001**		**0.001**		**0.036**
Single	6,591 (27.3)	174 (2.6)		33 (0.5)		42 (0.6)	
Married-monogamous	12,571 (52.2)	430 (3.4)		106 (0.8)		129 (1.0)	
Married-polygamous	4,584 (19.0)	186 (4.1)		54 (1.2)		46 (1.0)	
Other^**b**^	361 (1.5)	5 (1.4)		3 (0.8)		5 (1.4)	
**Education**			0.056		0.245		0.850
None/primary education (0–6)	10,746 (44.6)	370 (3.4)		88 (0.8)		102 (1.0)	
Secondary education (7–12+)	11,293 (46.8)	375 (3.3)		85 (0.8)		103 (0.9)	
NA	2,068 (8.6)	50 (2.4)		23 (1.1)		17 (0.8)	
**Parity**^**c**^			**< 0.001**		**< 0.001**		**< 0.001**
1	8,966 (37.2)	191 (2.1)		31 (0.4)		42 (0.5)	
2	5,534 (23.0)	205 (3.7)		48 (0.9)		55 (1.0)	
≥ 3	9,136 (37.9)	391 (4.3)		113 (1.2)		117 (1.3)	
Unknown	471 (2.0)	8 (1.7)		4 (0.9)		8 (1.7)	
**Year of delivery**			**0.024**		**0.017**		**< 0.001**
2008 (June–December)	1,514 (6.3)	46 (3.0)		9 (0.6)		23 (1.5)	
2009	4,801 (19.9)	191 (4.0)		56 (1.2)		74 (1.5)	
2010	6,534 (27.1)	220 (3.4)		52 (0.8)		58 (0.9)	
2011	3,901 (16.2)	126 (3.2)		24 (0.6)		27 (0.7)	
2012	5,336 (22.1)	162 (3.0)		46 (0.9)		29 (0.5)	
2013 (January–May)	2,021 (8.4)	50 (2.5)		9 (0.5)		11 (0.5)	

*p*-values < 0.05 highlighted in bold.

NA, Not available; *p*, *p*-value.

^a^Ethnic groups or nationalities, each comprising less than 1% of the sample population i.e., Cape Verdean, Senegalese, Guinean (Republic of Guinea), Balanta Mane, Mansoanca, Nalu, and Geba.

^b^Including widowed, divorced or separated.

^c^Including index pregnancy.

### Prevalence of HIV

A total of 5.1% (1,232/24,107) of women were infected with HIV. The overall prevalence of HIV-1 was 3.3% (95% CI 3.1–3.5), the HIV-2 prevalence was 0.8% (95% CI 0.7–0.9), and HIV-1/2 dually infected women accounted for 0.9% (95% CI 0.8–1.1). Nineteen women who tested positive did not have the HIV type discriminatory test performed (Fig. 1).

Among women tested at birth, 47% (11,233/24,107) reported prior HIV testing during antenatal visits. Among them, 5.0% (566/11,233) were HIV positive, 93% (10,444/11,233) were HIV negative, 0.1% (9/11,233) were indeterminate and 1.9% (214/11,233) could not remember. Comparing self-reported and observed HIV test results, we found that 3.4% (19/566) of women with self-reported HIV-positive status tested HIV negative in labour, and 1.9% (197/10,444) of self-reported HIV negative women were HIV positive at delivery. Of the women who could not remember their HIV status, 15% (32/214) were HIV positive.

### Variations in HIV-1 and HIV-2 prevalence

**Figure 2:**
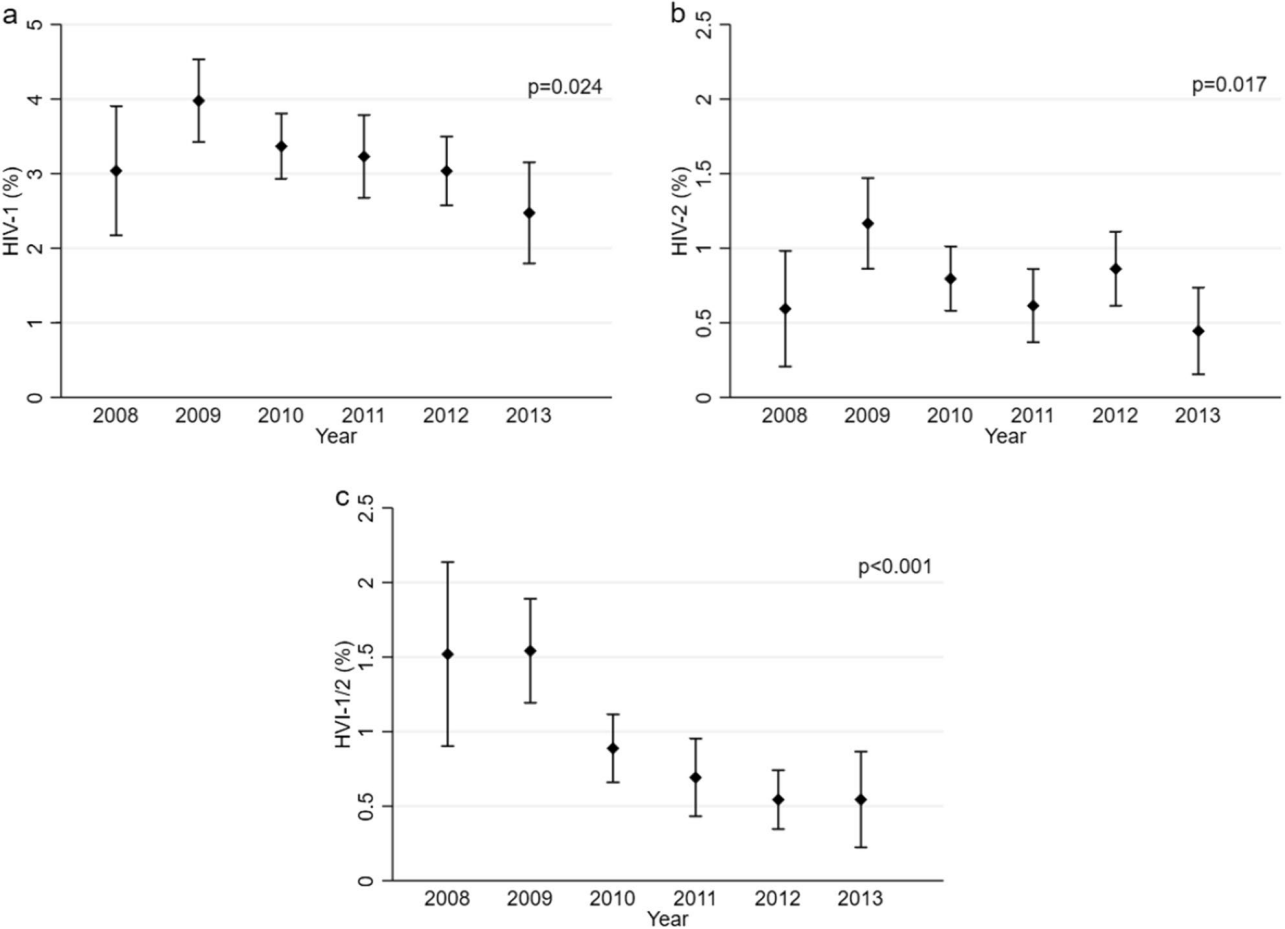
Prevalence of HIV-1 and HIV-2 in pregnant women by calendar year, Simão Mendes National Hospital, Bissau, Guinea-Bissau, 2008–2013. The figure displays the changes in HIV-1 (**a**), HIV-2 (**b**), and HIV-1/2 (**c**) prevalence (point estimates and corresponding 95% confidence intervals) among pregnant women presenting for birth by calendar year. HIV-1, HIV-2 and HIV-1/2 all declined significantly from 2009 to 2013 (chi^2^ test for trend).

There was a significant decline in HIV-1 prevalence from 4.0% (95% CI 3.5–4.6) in 2009 to 2.5% (95% CI 1.8–3.3) in 2013, *p* < 0.024. HIV-2 prevalence declined from 1.2% (95% CI 0.9–1.5) in 2009 to 0.5% (95% CI 0.2–0.8) in 2013, *p* < 0.017 (Fig. 2). Exploring changes in HIV prevalence by age groups, we observed that the decline in HIV-1 and HIV-1/2 prevalence was largely confined to older women (aged ≥ 25 years), while the decline in HIV-2 prevalence was independent of age (Supplemental Figure 1).

### Risk determinants of HIV-1 and HIV-2 infection

Based on univariate analysis, we found maternal age, marital status, parity, and calendar year were associated with HIV-1, HIV-2 and HIV-1/2. Surprisingly, education was not significantly associated with HIV status and was therefore excluded from the multivariate analysis. As expected, the risk of HIV-1 increased significantly with age in the multivariate model (Table 2). Women belonging to the Balanta [adjusted odds ratio [AOR] 1.41 (95% CI 1.09–1.82)], Fula [1.33 (95% CI 1.02–1.73)] and Mandinga [1.37 (95% CI 1.01–1.86)] ethnic groups, and women from smaller ethnic groups or non-Guinean heritage [2.08 (95% CI 1.60–2.70)], had a higher HIV-1 prevalence compared with the Pepel ethnic group. For HIV-2, only age was significantly associated with HIV infection. Lastly, we found age, the Mandinga ethnicity and calendar year were associated with HIV-1/2 (Table 2).

**Table 2:** Risk factors for HIV-1, HIV-2, and HIV-1/2 dual infection among all women tested for HIV at labour and delivery.

	HIV-1 positive n = 795			HIV-2 positive n = 196			HIV-1/2 positive n = 222		
	Coefficient ± SE	AOR (95% CI)^a^	*p*	Coefficient ± SE	AOR (95% CI)^a^	*p*	Coefficient ± SE	AOR (95% CI)^a^	*p*
**Age groups**									
Age 13–19		1.00			1.00			1.00	
Age 20–24	0.94 ± 0.15	**2.55 (1.91–3.40)**	**< 0.001**	0.82 ± 0.36	**2.27 (1.12–4.59)**	**0.022**	1.29 ± 0.34	**3.65 (1.86–7.14)**	**< 0.001**
Age 25–29	1.21 ± 0.16	**3.35 (2.47–4.54)**	**< 0.001**	1.41 ± 0.36	**4.10 (2.01–8.36)**	**< 0.001**	1.80 ± 0.35	**6.04 (3.04–12.01)**	**< 0.001**
Age 30+	1.25 ± 0.17	**3.50 (2.52–4.85)**	**< 0.001**	1.91 ± 0.38	**6.77 (3.24–14.13)**	**< 0.001**	1.99 ± 0.37	**7.34 (3.58–15.05)**	**< 0.001**
**Ethnic group**									
Balanta	0.34 ± 0.13	**1.41 (1.09–1.82)**	**0.009**	0.01 ± 0.24	1.01 (0.62–1.62)	0.984	− 0.01 ± 0.26	0.99 (0.60–1.65)	0.977
Bijagos	− 0.26 ± 0.35	0.77 (0.39–1.54)	0.463	− 0.22 ± 0.61	0.80 (0.24–2.65)	0.719	0.20 ± 0.54	1.22 (0.42–3.51)	0.718
Felupe	− 0.35 ± 0.40	0.71 (0.32–1.54)	0.381	0.01 ± 0.61	1.00 (0.30–3.33)	0.992	− 1.06 ± 1.02	0.35 (0.05–2.58)	0.302
Fula	0.28 ± 0.14	**1.33 (1.02–1.73)**	**0.038**	0.03 ± 0.25	1.03 (0.63–1.70)	0.905	0.35 ± 0.25	1.42 (0.87–2.32)	0.167
Mancanha	0.19 ± 0.17	1.21 (0.87–1.68)	0.253	− 0.09 ± 0.32	0.91 (0.49–1.71)	0.775	0.29 ± 0.30	1.34 (0.74–2.40)	0.336
Mandinga	0.32 ± 0.16	**1.37 (1.01–1.86)**	**0.041**	0.33 ± 0.27	1.39 (0.82–2.36)	0.227	0.82 ± 0.26	**2.28 (1.38–3.77)**	**0.001**
Manjaco	0.08 ± 0.17	1.08 (0.77–1.52)	0.658	0.02 ± 0.31	1.02 (0.56–1.87)	0.945	0.22 ± 0.30	1.25 (0.69–2.27)	0.461
Mixed ethnicity	0.17 ± 0.24	1.19 (0.75–1.90)	0.465	0.34 ± 0.39	1.41 (0.66–3.01)	0.373	0.46 ± 0.39	1.58 (0.74–3.41)	0.240
Pepel		1.00			1.00			1.00	
Saracule	− 0.10 ± 0.43	0.70 (0.30–1.60)	0.818	0.39 ± 0.62	1.48 (0.45–4.95)	0.521	–	–	–
Others^b^	0.80 ± 0.15	**2.08 (1.60–2.70)**	**< 0.001**	− 0.35 ± 0.36	0.70 (0.35–1.41)	0.322	0.40 ± 0.30	1.50 (0.84–2.68)	0.173
NA	1.29 ± 0.76	2.78 (0.63–12.20)	0.089		–		–	–	
**Marital status**									
Single		1.00			1.00			1.00	
Married-monogamous	− 0.12 ± 0.10	0.89 (0.72–1.08)	0.238	− 0.09 ± 0.22	0.92 (0.59–1.42)	0.696	− 0.09 ± 0.20	0.92 (0.62–1.36)	0.668
Married-polygamous	0.00 ± 0.12	1.00 (0.79–1.27)	0.999	0.11 ± 0.25	1.11 (0.68–1.83)	0.678	− 0.18 ± 0.20	0.84 (0.52–1.35)	0.473
Other^c^	− 0.76 ± 0.68	0.47 (0.12–1.78)	0.2634	− 0.13 ± 1.03	0.88 (0.12–6.53)	0.897	− 0.80 ± 0.74	0.45 (0.11–1.91)	0.277
**Parity**									
1^d^		1.00			1.00			1.00	
2	0.22 ± 0.11	1.25 (1.00–1.55)	0.052	0.45 ± 0.25	1.57 (0.96–2.58)	0.076	0.27 ± 0.22	1.30 (0.84–2.02)	0.234
≥ 3	0.19 ± 0.12	1.21 (0.96–1.52)	0.113	0.37 ± 0.26	1.44 (0.86–2.40)	0.161	0.24 ± 0.23	1.27 (0.81–1.98)	0.300
NA	− 0.08 ± 0.55	0.93 (0.32–2.70)	0.890	0.39 ± 0.90	1.48 (0.25–8.60)	0.665	1.34 ± 0.60	**3.80 (1.17–12-35)**	**0.026**
**Year**									
2008 (June–December)		1.00			1.00			1.00	
2009	0.25 ± 0.17	1.29 (0.92–1.79)	0.136	0.63 ± 0.36	1.87 (0.92–3.80)	0.084	− 0.01 ± 0.24	0.99 (0.62–1.60)	0.978
2010	0.07 ± 0.17	1.07 (0.77–1.48)	0.693	0.21 ± 0.36	1.24 (0.61–2.53)	0.561	− 0.57 ± 0.25	0.56 (0.35–0.92)	0.022
2011	0.01 ± 0.18	1.01 (0.72–1.43)	0.943	− 0.06 ± 0.39	0.94 (0.43–2.03)	0.872	− 0.84 ± 0.29	**0.43 (0.25–0.76)**	**0.003**
2012	− 0.07 ± 0.17	0.93 (0.67–1.31)	0.687	0.28 ± 0.37	1.32 (0.64–2.71)	0.450	− 1.11 ± 0.28	**0.33 (0.19–0.57)**	**< 0.001**
2013 (January–May)	− 0.28 ± 0.21	0.76 (0.50–1.14)	0.177	− 0.42 ± 0.48	0.66 (0.26–1.66)	0.375	− 1.13 ± 0.37	**0.33 (0.16–0.67)**	**0.002**
Constant	− 4.71 ± 0.22	**–**		− 6.58 ± 0.49	**–**		− 6.00 ± 0.42	–	

*p*-values < 0.05 highlighted in bold.

NA, Not available; SE, Standard error; AOR, adjusted odds ratio; CI, confidence intervals; *p*, *p*-value.

^a^Variables associated with HIV-1, HIV-2 or HIV-1/2 infection in the univariate analysis (*p* < 0.05) were included in the multivariate model.

^b^Ethnic groups or nationalities, each comprising less than 1% of the sample population i.e., Cape Verdean, Senegalese, Guinean (Republic of Guinea), Balanta Mane, Mansoanca, Nalu, and Geba.

^c^Separated, divorced or widowed.

^d^Including index pregnancy.

### ART and PMTCT regimens

**Figure 3:**
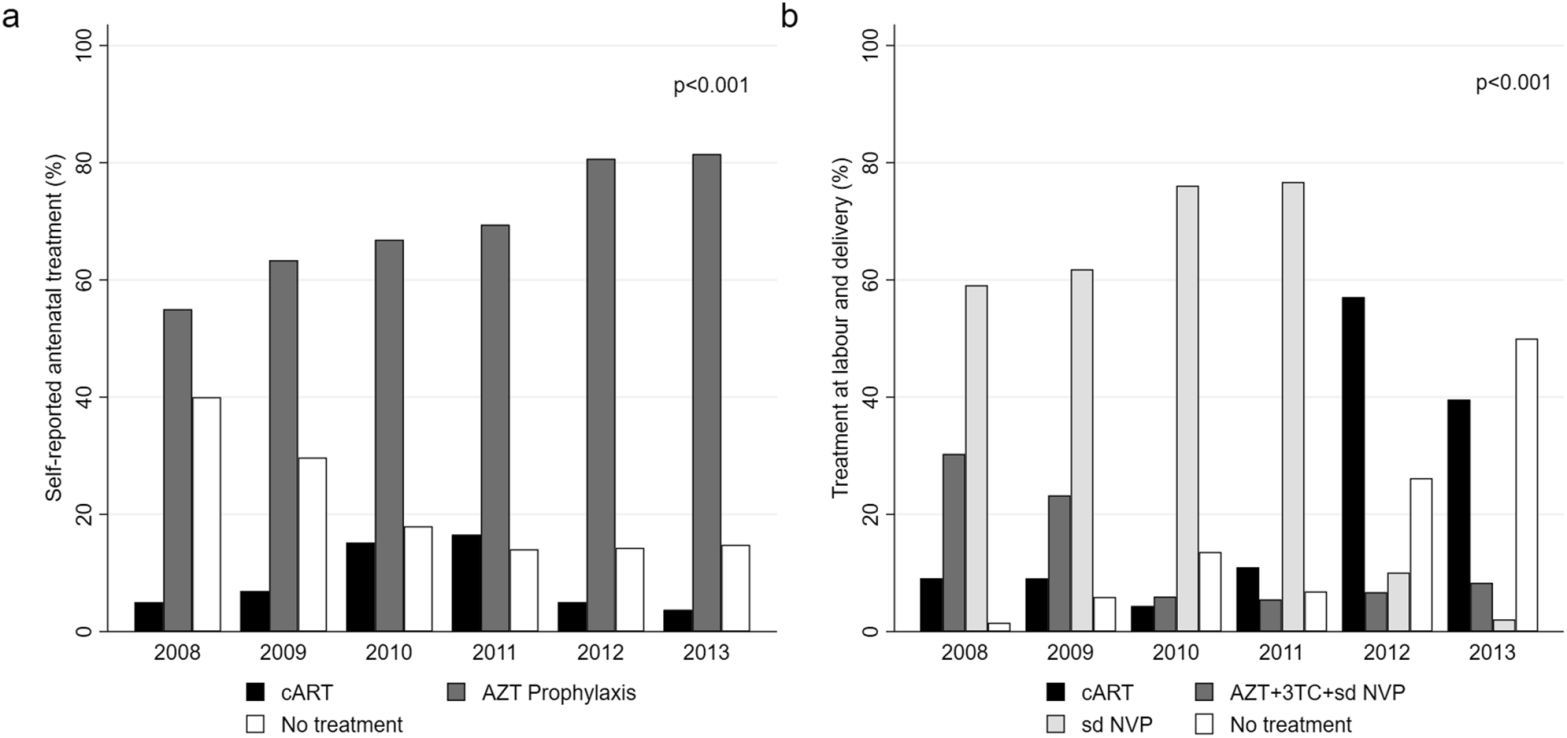
Self-reported antenatal treatment (**a**) and treatment provided at birth (**b**) by calendar year, Simão Mendes National Hospital, Bissau, Guinea-Bissau, 2008–2013. *cART* combined antiretroviral therapy, *AZT* Zidovudine, *3TC* Lamivudine, *sd NVP* single-dose Nevirapine. (**a**) Self-reported treatment regimens received by HIV-positive women presenting for labour by calendar year (N = 566). As shown, the percentage of women receiving AZT prophylaxis increased between 2008 and 2013, while the percentage of women on cART increased slightly from 2008 to 2011 and declined thereafter. (**b**) The provision of antiretroviral treatment at birth by calendar year, excluding women who reported initiating ART during the antenatal period (N = 774). The figure shows that the proportion of women receiving combined antiretroviral treatment increased steadily between 2009 and 2012, yet the percentage decreased between 2012 and the first quarter of 2013. Conversely, a decline in women receiving option A [AZT prophylaxis + (AZT + 3TC and sd NVP at labour)] between 2008 and 2013 was observed. Women receiving only sd NVP declined notably, suggesting that more women were initiated on treatment before labour. During the same period, we observed a notable increase in the number of HIV-infected women not receiving treatment at delivery. The *p *values shown were estimated using the chi^2^ test for trend.

**Figure 4:**
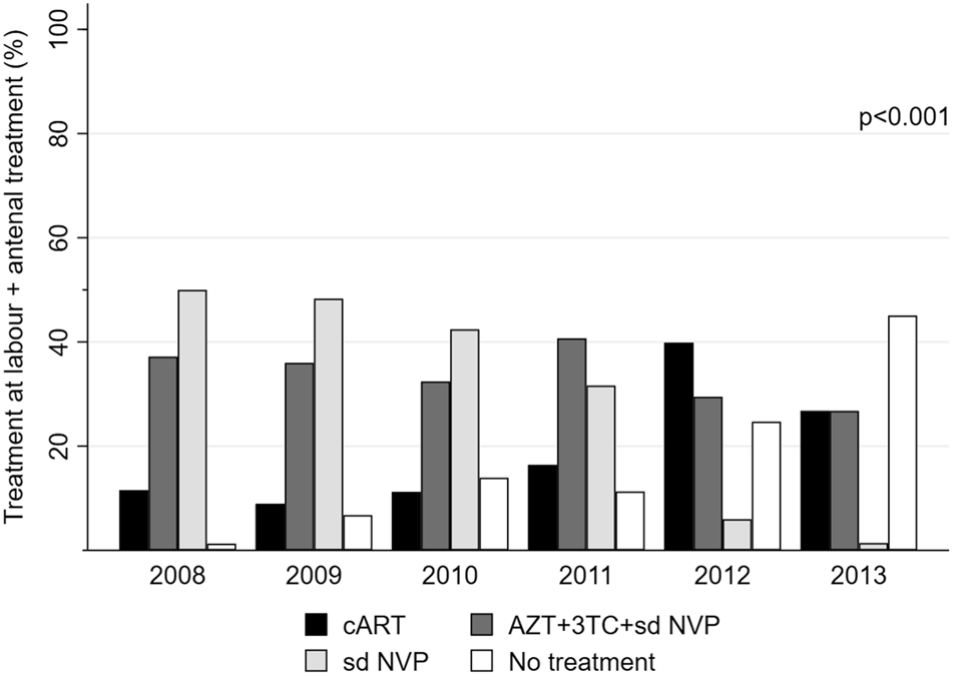
The provision of antiretroviral treatment for all HIV-infected women by calendar year, Simão Mendes National Hospital, Bissau, Guinea-Bissau, 2008–2013. *cART* combined antiretroviral therapy, *AZT* Zidovudine, *3TC* Lamivudine, *sd NVP* single-dose Nevirapine. The figure displays the provision of antiretroviral treatment at birth by calendar year for all HIV women in this study (N = 1,232). Overall coverage for cART and option A (AZT + 3TC + sd NVP) remained relatively low throughout the study period. There was a decline in the number of women receiving sd NVP, while the overall number of women not receiving any treatment increased over time. The *p *value shown was estimated using the chi^2^ test for trend.

Of women diagnosed antenatally (N = 566), 70% (396/566) reported receiving AZT prophylaxis, while 11% (62/566) reported receiving cART. Nineteen percent (108/566) reported that they were not receiving treatment or could not remember. Among women diagnosed in labour and women reporting no antenatal ART, 19% (149/774) received cART, 14% (108/774) received AZT prophylaxis plus a sd NVP + initiation of 3TC + AZT at childbirth, while 52% (408/774) of women, first diagnosed at delivery, received only sd NVP. Fourteen percent (109/774) did not receive any treatment at delivery. For women with HIV-2 (n = 196), 39% (76/196) received prophylaxis AZT plus a single dose-NVP + initiation of 3TC + AZT at childbirth, while 28% (55/196) received sd NVP only. Among women aware of their HIV status prior to labour and delivery, we observed a significant increase in self-reported antenatal treatment coverage from 60% in 2008 to 85% in 2013, *p* < 0.001. Specifically, there was an increase in women receiving antenatal AZT prophylaxis from 55% in 2008 to 82% in 2013, while there was no overall increase in cART during the same period (Fig. 3a). For women diagnosed at delivery and HIV-positive women not receiving antenatal ART (excluding women already on treatment, n = 458), overall treatment coverage declined from 99% in 2008 to 50% in 2013, *p* < 0.001. Notably, the proportion of women not receiving treatment at birth increased steadily throughout the study period (Fig. 3b). Figure 3 shows the provision of self-reported antenatal treatment and treatment at birth among HIV-infected women at the HNSM. During the study period, 85% of all HIV-infected women (1,049/1,232) received some form of ART in relation to labour for PMTCT. The majority (34%, n = 418) received option A, 33% (n = 408) received sd NVP, 18% (n = 223) received triple ART and 15% (n = 183) did not receive any treatment. As with women diagnosed at delivery, we observed a steady increase in the number of women not receiving any treatment despite including women diagnosed antenatally (Fig. 4).

Among infants of HIV-infected mothers (including twins and triplets), 8% (103/1,275) received the recommended treatment of sd NVP followed by AZT treatment, 70% (891/1,275) received sd NVP, and 22% (281/1,275) did not receive any treatment. Among infants of HIV-2 infected mothers, 77% (156/202) received sd NVP or sd NVP followed by AZT. The number of infants not receiving treatment to prevent mother-to-child transmission increased from 7% in 2008 to 36% in 2013, *p* < 0.001 (Supplemental Figure 2).

### Birth outcomes

Seventeen percent (4,213/25,232) of births were LBW, and 10% (2,513/25,256) of babies were stillborn. Factors independently associated with LBW and stillbirths are displayed in Supplemental Table 1. We found no association between antenatal treatment and birth outcomes. Assessing birth outcomes for HIV-1, HIV-2 and HIV-1/2 dually infected women compared with HIV negative women, we found that maternal HIV-1 and HIV-1/2 co-infection were associated with LBW, AOR 1.25 (95% CI 1.03–1.52) and AOR 1.73 (95% CI 1.26–2.37), respectively (Table 3). No significant association was found between HIV status and stillbirth in univariate or multivariate analyses (Table 4).

**Table 3:** Crude and adjusted odds ratios for low birth weight according to HIV status among mothers presenting for labour in Bissau, Guinea-Bissau (N = 25,232).

Maternal HIV status (N = 24,107)		Low birth weight (BW < 2,500 g)^a^ (N = 25,232)						
	n (col%)	Yes n (row%)	Coefficient ± SE	COR (95% CI)	*p*	Coefficient ± SE	AOR (95% CI)^b^	*p*
Negative	22,894 (95.0%)	3,991 (17.4%)		1.00			1.00	
HIV-1	795 (3.3%)	140 (17.6%)	0.05 ± 0.10	1.05 (0.87–1.26)	0.604	0.22 ± 0.10	**1.25 (1.03–1.52)**	**0.027**
HIV-2	196 (0.8 %)	28 (14.3%)	− 0.20 ± 0.21	0.82 (0.55–1.22)	0.320	− 0.03 ± 0.22	0.97 (0.64–1.48)	0.882
HIV-1/2	222 (0.9%)	54 (24.3%)	0.42 ± 0.16	**1.52 (1.12–2.07)**	**0.007**	0.55 ± 0.16	**1.73 (1.26–2.37)**	**0.001**
Constant			− 1.61 ± 0.02			− 2.26 ± 0.22		

*p*-values < 0.05 highlighted in bold.

BW, birth weight; SE, Standard error; COR, crude odds ratio; CI, confidence intervals; AOR, adjusted odds ratio; *p*, *p*-value.

^a^Including twin and triplet births.

^b^Adjusted for age by groups, ethnicity, marital status, education, parity, vital status of last-born child, previous antenatal counselling, Bissau resident (resident or referral patient from another region) and twin birth.

**Table 4:** Crude and adjusted odds ratios for stillbirth according to HIV status among mothers presenting for labour in Bissau, Guinea-Bissau (N = 25,256).

Maternal HIV status (N = 24,107)		Stillbirth (N = 25,256)^a^						
	n (col%)	Yes n (%)	Coefficient ± SE	COR (95% CI)	*p*	Coefficient ± SE	AOR (95% CI)^b^	*p*
Negative	22,894 (95.0%)	2,382 (10.4%)		1.00			1.00	
HIV-1	795 (3.3%)	81 (10.2%)	0.01 ± 0.12	1.01 (0.80–1.28)	0.930	0.05 ± 0.13	1.05 (0.82–1.36)	0.692
HIV-2	196 (0.8%)	22 (11.2%)	0.12 ± 0.23	1.12 (0.72–1.75)	0.611	0.04 ± 0.25	1.00 (0.62–1.63)	0.988
HIV-1/2	222 (0.9%)	28 (12.6%)	0.22 ± 0.20	1.25 (0.84–1.85)	0.277	0.01 ± 0.22	1.01 (0.65–1.56)	0.958
Constant			− 2.21 ± 0.02			− 4.40 ± 0.22		

SE, Standard error; COR, crude odds ratio; CI, confidence intervals; AOR, adjusted odds ratio; *p*, *p*-value.

^a^Including twin and triplet births.

^b^Adjusted for age by groups, ethnicity, marital status, education, parity, caesarean-section, vital status of last-born child, previous antenatal counselling, Bissau resident (resident or referral patient from another region), low birth weight and twin birth.

## Discussion

This retrospective cross-sectional study examined HIV-1 and HIV-2 prevalence and factors associated with HIV, PMTCT treatment coverage and adverse birth outcomes at the largest maternity ward in Guinea-Bissau. It shows a decline in HIV-1, HIV-2, HIV-1/2 prevalence among pregnant women presenting for labour during a 5-year period. Women from the Balanta and Fula ethnic groups had a significantly higher risk of HIV-1. Antenatal treatment coverage appears to be improving over time, yet an increase in the proportion of mothers and infants not receiving treatment at birth was observed, inevitably increasing the overall risk of mother-to-child transmission. This is specifically alarming as nearly half of the women were unaware of their HIV status prior to presenting for labour. Over the 5-year period, 15% of HIV-infected mothers and 22% of infants born to HIV-infected mothers did not receive any treatment. In addition, 67% of mothers with HIV-2 received NVP, which is ineffective against HIV-2 infection. Lastly, infants born to mothers with HIV-1 or HIV-1/2 dually infected were at risk of being LBW.

The HIV-2 prevalence of 0.8% in this survey was notably lower than in previous surveys among pregnant women, suggesting a continuing decline in HIV-2 prevalence in Guinea-Bissau^8,24–26^. A national survey from 2013 conducted in all 10 regions of the country reported an HIV-2 (including HIV-1 co-infected) prevalence of 1.0% (95% CI 0.5–1.8)^27^. In addition, a recently published study from the Bandim Health Project demographic surveillance site found an HIV-2 prevalence of 1.5% among younger women (aged 15–44 years)^7^. Changes in risk behaviour over the past decades and competitive exclusion have been proposed as explanations^28,29^. Modelling predicts that new HIV-2 cases in the country will cease within a few decades ^30^. In contrast, previous surveys from Guinea-Bissau have shown increasing or stabilized HIV-1 prevalence among a range of populations^7,24,26,31^. Gianelli et al.^8^ found that HIV-1 prevalence among pregnant women stabilized between 2003 and 2006. Our study is supported by findings from a recent national survey suggesting that the HIV-1 prevalence has stabilized and may be declining among women of reproductive age^27^. The risk of HIV transmission decreases substantially with ART^32^, and the decline in HIV-1 and HIV-2 may in part reflect the fruits of national interventions, including efforts to strengthen prevention measures during the roll-out of ART. Nevertheless, inadequate health infrastructure, repeated drug stock outages, poor adherence, and high rates of loss to follow in Guinea-Bissau increase the risk of treatment failure^7,10,14,33–35^ and thus infectivity, which could reverse current encouraging trends in HIV prevalence. Sustained and sustainable long-term management of affected patients, combined with prevention, is required^10^.

The Mandinga and Fula, two important West African ethnic groups, had a significantly higher prevalence of HIV-1 compared with other ethnic groups. A study by Kenyon et al.^36^ has suggested that differences in sexual behaviour according to ethnic group may contribute to a large variation in HIV prevalence. Previous research from Guinea-Bissau has suggested that female genital mutilation and polygamy among these ethnicities may play a role^8,37,38^. Furthermore, a recent study from the HIV clinic at HNSM found Fula and Mandinga ethnicity to be associated with late presentation at HIV diagnosis^39^. These findings underline the importance of identifying vulnerable populations, understanding sociocultural motivators, and targeting interventions accordingly.

Overall, self-reported HIV treatment coverage among women accessing antenatal care increased significantly over the course of this study. However, only 47% of women presenting for labour had received antenatal testing and counselling, and only 77% of women were tested for HIV at birth, leaving a large number of women unaware and untreated^14^. HIV-infected women not identified during pregnancy miss out on the benefits of treatment and prevention and remain part of a “hidden epidemic”; they are prone to late diagnosis, poor health outcomes and at risk of infecting their infants^40,41^.

While some national interventions, such as strengthening the early provision of ART as recommended by the WHO, have shown promise^18^, repeated political instability, and inadequate management of HIV tests and medicine stocks appear to have significant negative impacts on the clinical management of HIV in the country^10,14,42^. After a civil war in 1999, Guinea-Bissau suffered five military coups between 2002 and 2012^43,44^, which left the country in a chronic state of political instability^14^. This has hampered development despite national and international efforts and inevitably discouraged donors, as was seen in 2012 after the Global Fund, the World Bank, the African Development Bank and the European Union suspended HIV funding activities in Guinea-Bissau^12,21^. These cuts in funding may explain the increase in HIV-infected women and their infants not receiving ART in labour in 2012 and 2013 as observed in this study, as well as the decline in national ART coverage from 56% in 2011 to 23% in 2014^21^. Repeated drug stock outages were common in Guinea-Bissau^9,10,42^ and may have forced doctors and midwives to choose treatments no longer recommended by national guidelines, as reflected by the high proportion of women receiving sd NVP, or no treatment. In addition, a high number of HIV-2-infected patients received NVP, a non-nucleoside reverse transcriptase inhibitor (NNRTI), for PMTCT. This is problematic because NNRTIs are ineffective against HIV-2 due to natural resistance^45^. Periods of drug shortages and the resulting use of NNRTI-based regimes may explain the high levels of NNRTI resistance found in Guinea-Bissau^42,46,47^. Conditions in the country appear to have improved after international donors, led by the Global Fund, have resumed funding activities, which is reflected by a 30% increase (49.5–81.9%) in the number of HIV-positive mothers who received ART for PMTCT in 2014^21^. Current estimates from UNAIDS report that 65% of pregnant women living with HIV in Guinea-Bissau accessed ART in 2017^48^. While these results show promise, overall ART coverage remains low in Guinea-Bissau^48^. Preliminary results from a routine analysis of nationwide HIV Cohort in the country show that only 14% of HIV infected individuals are aware of their HIV-status and of these only 20% are on treatment underling the urgent need to determine the treatment retention and long-term outcomes of mothers and their infants during and after PMTCT^10^.

This study found an increase in the number of infants not receiving PMTCT. An increase in the number of HIV-infected children and high rates of HIV infection (9.3%) despite PMTCT have been reported in Guinea-Bissau, suggesting poor treatment retention and challenges to PMTCT coverage and follow-up^15^. While an increase in the number of HIV-infected children may be due to better diagnostics, the late diagnosis of children (median age 3.5 years) suggests a large proportion of children die before being diagnosed with HIV^15^.

In line with other studies, women infected with HIV-1 and HIV-1/2 in this study had an increased risk of delivering LBW babies^49,50^. LBW babies have an increased risk of mortality and may experience negative health outcomes during childhood and later in life^51^. Studies have described an increasing risk of LBW with advancing stage of HIV disease^52^, but also according to maternal ART regimens^51^, emphasizing the importance of identifying HIV-infected mothers early in pregnancy and insuring they receive the optimal ART regimen to improve health outcomes, prevent MTCT and minimize the risk of LBW.

In Guinea-Bissau, stillbirths are common. A previous study from the capital Bissau found a community stillbirth rate of 71/1,000 among mothers who did not attend ANC and 36/1,000 among mothers who attended 7 or more ANC^16^. While HIV infection is known to be associated with stillbirths^50^, this study did not find a significant association. Many factors affect the risk of stillbirths, including maternal age, education, marital status and ethnicity, in our setting^16^. In addition, the stage of maternal disease, ART regimen and timing of treatment play a role in the risk of stillbirth and other adverse birth outcomes among HIV-infected mothers^4,53^. Therefore, our analysis may have been limited by our inability to control sufficiently for these factors.

This study is based on data from a large sample of women presenting for labour and delivery at the country’s largest maternity ward and thus reflects the challenges to PMTCT services in a setting with limited resources. Our research has several limitations. First, the cross-sectional nature of this study means we are unable to determine trends in incidence and causality. Twenty-three percent of women presenting for labour during the study period were not counselled or tested for HIV. These women were not included in this study due to missing information on HIV status presenting a potential selection bias. Data regarding condom use, number of previous partners, and additional high-risk behaviours were unavailable, limiting our risk factor analysis. HIV type discrimination was performed using SD Bioline HIV-1/2 3.0. A study from Guinea-Bissau found that SD Bioline HIV-1/2 3.0 overestimates the number of HIV-1/2 dually infected individuals^54^, and high inter-observer variation was noted^55^, which could have led to misclassification of HIV type in this study. HIV prevalence was determined based on women presenting at the HNSM maternity ward and may differ from women giving birth at other urban health centres or at home. Only half (47%) of women tested at birth reported previous testing during antenatal visits. There were discrepancies between self-reported antenatal test results and results at labour suggesting either insufficient pre and post testing counselling, lack of HIV awareness, or stigma^13^, recall bias or seroconversion.

The HNSM maternity ward receives women from all parts of the capital and other regions in Guinea-Bissau for care and treatment of obstetric complications, yet little is known regarding the specific referral process to give birth. The lack of continuity of care and registration systems made it almost impossible for midwives to corroborate self-reported antenatal treatment, which may have been affected by recall bias. In addition, the lack of confidentiality and time to perform pre- and post-test counselling at labour^13^ could have limited midwives’ and doctors’ ability to make treatment decisions regarding the type and length of treatment based on self-reported antenatal treatment regimes.

## Conclusion

The prevalence of HIV-1, HIV-2 and HIV-1/2 among pregnant women presenting for labour and delivery at the HNSM declined between 2008 and 2013. The observed increase in the proportion of women and infants not receiving treatment at birth is concerning. Less than half of the women in this study reported attending antenatal counselling and testing, and 23% were not tested in relation at birth, resulting in many mothers unaware of their HIV status and hence deprived of the benefits of treatment and prevention. As Guinea-Bissau continues to take strides towards improving PMTCT testing and ART coverage to mitigate the risk of poor health outcomes of HIV-infected mothers and their infants, further operational research focusing on provision of PMTCT services and continuity of care, retention to PMTCT services, including treatment and patient follow-up, is urgently needed to support future interventions and policy.

## Data availability

The original data are stored at the Bandim Health Project, National Institute of Public Health in Guinea-Bissau. The ethical approvals received from The National Committee on Ethics in Health, National Institute of Public Health, Ministry of Public Health, Guinea-Bissau (e-mail: inasagb@gmail.com) do not allow for general data sharing of this dataset. Data access can therefore be granted to individual researchers only after obtaining ethical permission. Any party interested in accessing the relevant data can do so by contacting the Bandim Health Project (e-mail: bandim@ssi.dk). The Bandim Health Project will facilitate the request for ethical permission from the National Committee on Ethics in Health and provide the data on approval. We can confirm that others will be able to access the relevant data in the same manner and form as the authors, provided ethical approval is obtained from Guinea-Bissau’s National Committee on Ethics in Health.

## Supplementary information

Supplementary Information 1.

Supplementary Information 2.
