# Supplementary material for: HIV-1 and HIV-2 prevalence, risk factors and birth outcomes among pregnant women in Bissau, Guinea-Bissau: a retrospective cross-sectional hospital study
Source: Sci Rep. 2020 Jul 22;10:12174. doi: 10.1038/s41598-020-68806-5 (PMC7376101; doi:10.1038/s41598-020-68806-5)
Supplement: Supplementary file 1 — Supplementary Information 1. [file 41598_2020_68806_MOESM1_ESM.docx]

**HIV-1 and HIV-2 prevalence, risk factors and birth outcomes among pregnant women in Bissau, Guinea-Bissau: a retrospective cross-sectional hospital study**

**Authors:** Dlama Nggida Rasmussen^1,2,3*^ Noel Vieira^4^, Bo Langhoff Hønge^3,5,6^, David da Silva Té^7^, Sanne Jespersen^3,6^, Morten Bjerregaard-Andersen^3,8,9^, Inés Oliveira^3^, Alcino Furtado^3^, Magarida Alfredo Gomes^10^, Morten Sodemann^2,3^, Christian Wejse^3,6,11^, Holger Werner Unger^3,12,13^

**Affiliations:**

^1^ Department of Public Health, University of Southern Denmark, Odense, Denmark.

^2^ Department of Infectious Diseases, Odense University Hospital, Odense, Denmark.

^3^ Bandim Health Project, INDEPTH Network, Bissau, Guinea-Bissau.

^4^ Association Ceu e Terras, Bissau, Guinea-Bissau.

^5^ Department of Clinical Immunology, Aarhus University Hospital, Aarhus, Denmark.

^6^ Department of Infectious Diseases, Aarhus University Hospital, Aarhus, Denmark.

^7^ National HIV Programme, Secretariado Nacional de Luta Contra le Sida, Ministry of Health, Guinea-Bissau.

^8^ Department of Endocrinology, Hospital of South West Denmark, Esbjerg, Denmark.

^9^ Research Center for Vitamins and Vaccines, Statens Serum Institut, Copenhagen, Denmark.

^10^ Department of Obstetrics and Gynaecology, Simão Mendes National Hospital, Bissau, Guinea-Bissau.

^11^GloHAU, Center for Global Health, Department of Public Health, Aarhus University, Aarhus, Denmark.

^12^ Centre for Maternal and Newborn Health, Liverpool School of Tropical Medicine, Liverpool, United Kingdom.

^13^ Department of Medicine at the Doherty Institute, The University of Melbourne, Australia.

**Corresponding author:** Dlama Nggida Rasmussen, Department of Public Health, University of Southern Denmark, Winsløwparken 19.2, DK-5000 Odense, Denmark.

E-mail: [drasmussen@health.sdu.dk](mailto:drasmussen@health.sdu.dk) Phone: 0045 3137 2992

**Supplementary information**

**Table legends.**

Table 1.

**Legend.** NA, Not available; *p*, p-value.

**^A^** Ethnic groups or nationalities, each comprising less than 1% of the sample population i.e., Cape Verdean, Senegalese, Guinean (Republic of Guinea), Balanta Mane, Mansoanca, Nalu, and Geba.

**^B^** Including widowed, divorced or separated.

**^C^** Including index pregnancy.

Table 2.

**Legend.** NA, Not available; SE, Standard error; AOR, adjusted odds ratio; CI, confidence intervals; p, p-value.

^A^ Variables associated with HIV-1 or HIV-2 infection in the univariate analysis (p<0.05) and included in a multivariate model.

^B^ Ethnic groups or nationalities, each comprising less than 1% of the sample population i.e., Cape Verdean, Senegalese, Guinean (Republic of Guinea), Balanta Mane, Mansoanca, Nalu, and Geba.

^C^ Separated, divorced or widowed.

^D^ Including index pregnancy.

Table 3.

**Legend.** BW, birth weight; SE, Standard error; COR, crude odds ratio; CI, confidence intervals; AOR, adjusted odds ratio; *p*, p-value.

^A^ Including twin and triplet births.

^B^ Adjusted for age by groups, ethnicity, marital status, education, parity, vital status of last-born child, previous antenatal counselling, Bissau resident (resident or referral patient from another region) and twin birth.

Table 4.

**Legend.** SE, Standard error; COR, crude odds ratio; CI, confidence intervals; AOR, adjusted odds ratio; *p*, p-value.

^A^ Including twin and triplet births.

^B^ Adjusted for age by groups, ethnicity, marital status, education, parity, caesarean-section, vital status of last-born child, previous antenatal counselling, Bissau resident (resident or referral patient from another region), low birth weight and twin birth.

**S1 Figure.** The point prevalence of HIV-1(a) and HIV-2 (b) in pregnant women according to age groups and by calendar year.


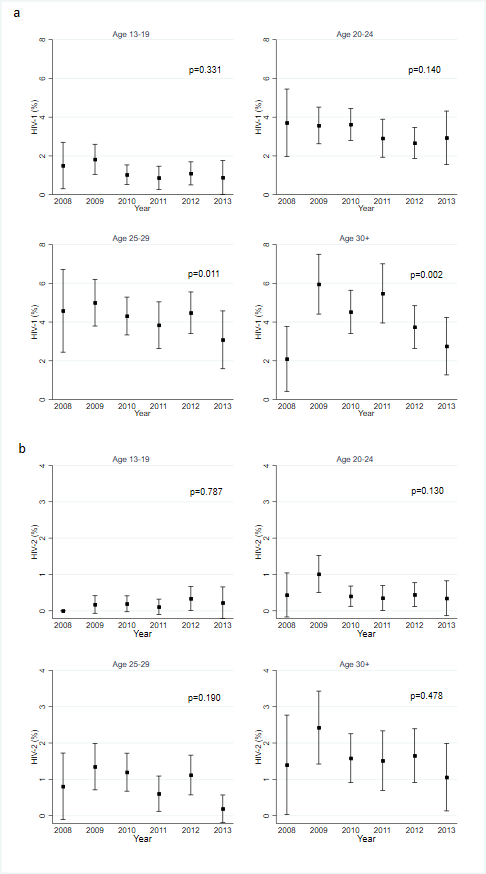

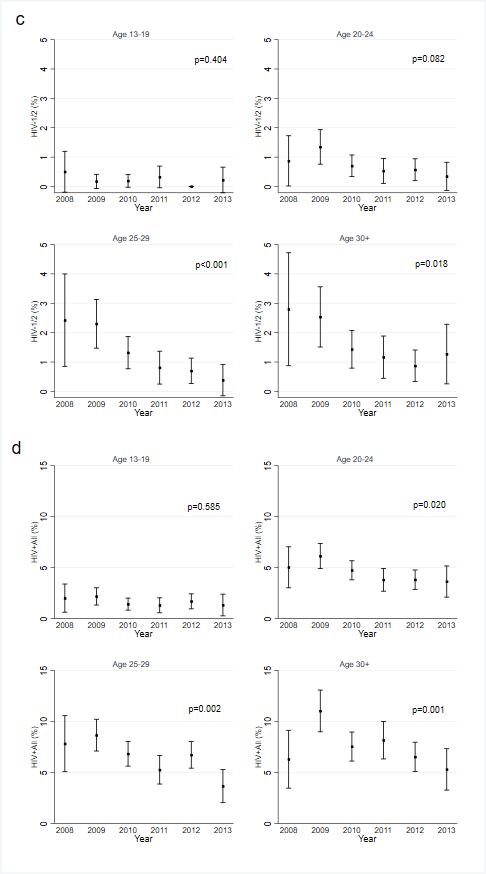


**Legend.** Figures **a**, **b, c and d** show the changes in HIV point prevalence stratified by age and calendar year.

a) For HIV-1 and HIV-1/2 infected we observe a significant decline in the HIV prevalence among women age 25-29 years and age 30+ years. b) Among HIV-2 infected the HIV prevalence did not decline significantly according to age groups. Finally, the overall HIV prevalence declined significantly for all age groups except women aged 13-19. Point estimates are displayed with corresponding 95% confidence intervals. P values were estimated using chi^2^ test for trend.

**S2 Figure**. The provision of infant antiretroviral prophylaxis by calendar year, Simão Mendes National Hospital, Bissau, Guinea-Bissau, 2008 – 2013 (N=1,275).


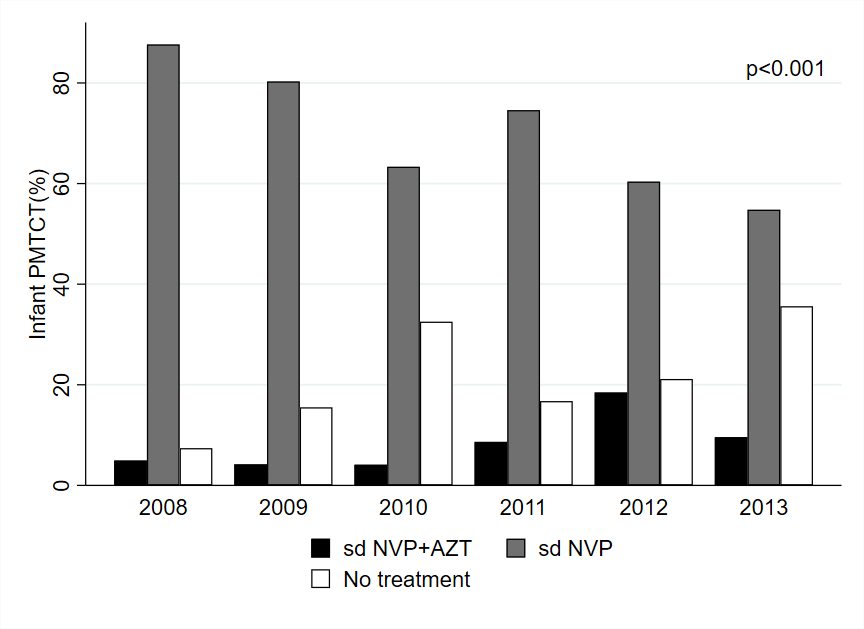


**Legend.**  sd NVP, single-dose Nevirapine; AZT, Zidovudine.

The figure displays the provision of infant antiretroviral treatment with either sd NVP followed by AZT twice daily (n=103) or only sd NVP(n=891). As displayed the percentage of infants receiving sd NVP declined between 2008 and 2013, while the percentage of children not receiving treatment at labour increased significantly during the same period (chi^2^ test for trend, p< 0.001). The recommended regime of sd NVP+AZT increased slightly from 2008 to 2012 after which a decline was observed.

**S1 Table**. Correlates of birth outcomes (low birth weight and stillbirths) according to maternal and infant characteristics in Bissau, Guinea-Bissau.

| Characteristic | | | Study population  N=24,107 | Low birth weight (BW<2500g)  n=25,232^G,H^ | | | Stillbirth  n=25,256^H,I^ | | |
| --- | --- | --- | --- | --- | --- | --- | --- | --- | --- |
|  | | | n(col%) | No  n(row%) | Yes  n(row%) | *p* | No  n(row%) | Yes  n(row%) | *p* |
| **Maternal** | | |  |  |  |  |  |  |  |
| Age groups | | |  |  |  | **<0.001** |  |  | **<0.001** |
|  | Age 13-19 | | 5,655(23.5) | 4,664(81.2) | 1,082(18.8) |  | 5,203(90.5) | 545(9.5) |  |
|  | Age 20-24 | | 7,215(29.9) | 6,305(83.7) | 1,229(16.3) |  | 6,901(91.5) | 644(8.5) |  |
|  | Age 25-29 | | 6,238(25.9) | 5,570(84.2) | 1,049(15.9) |  | 5,984(90.4) | 638(9.6) |  |
|  | Age 30+ | | 4,999(20.7) | 4,480(84.0) | 853(16.0) |  | 4,655(87.2) | 686(12.8) |  |
| Ethnic group | | |  |  |  | **<0.001** |  |  | **<0.001** |
|  | Balanta | | 5,476(22.7) | 4,820(82.9) | 995(17.1) |  | 5,125(88.0) | 697(12.0) |  |
|  | Bijagos | | 436(1.8) | 408(89.9) | 46(10.1) |  | 409(90.1) | 45(9.9) |  |
|  | Felupe | | 374(1.6) | 341(87.0) | 51(13.0) |  | 361(91.9) | 32(8.1) |  |
|  | Fula | | 5,262(21.8) | 4,448(81.0) | 1,042(19.0) |  | 4,915(89.4) | 581(10.6) |  |
|  | Mancanha | | 1,878(7.8) | 1,657(85.3) | 286(14.7) |  | 1,826(93.9) | 118(6.1) |  |
|  | Mandinga | | 2,485(10.3) | 2,086(80.0) | 521(20.0) |  | 2,353(90.2) | 255(9.8) |  |
|  | Manjaco | | 1,860(7.7) | 1,648(85.2) | 287(14.8) |  | 1,791(92.5) | 145(7.5) |  |
|  | Mixed ethnicity | | 682(2.8) | 612(88.4) | 80(11.6) |  | 652(94.2) | 40(5.8) |  |
|  | Pepel | | 3,495(14.5) | 3,104(84.8) | 558(15.2) |  | 3,263(89.0) | 405(11.0) |  |
|  | Saracule | | 252(1.1) | 218(84.2) | 41(15.8) |  | 249(96.1) | 10(3.9) |  |
|  | Others ^A^ | | 1,886(7.8) | 1,654(84.4) | 305(15.6) |  | 1,779(90.8) | 181(9.2) |  |
|  | NA | | 21 (0.1) | 23(95.8) | 1(4.2) |  | 20(83.3) | 4(16.7) |  |
| Marital status | | |  |  |  | **<0.001** |  |  | **<0.001** |
|  | Single | | 6,407(26.6) | 5,439(83.3) | 1,090(16.7) |  | 6,009(92.0) | 526(8.0) |  |
|  | Married-monogamous | | 11,754(48.8) | 10,447(84.1) | 1,981(15.9) |  | 11,375(91.5) | 1,061(8.5) |  |
|  | Married-polygamous | | 5,633(23.4) | 4,939(82.6) | 1,041(17.4) |  | 5,210(87.1) | 775(13.0) |  |
|  | Others^C^ | | 313(1.3) | 194(65.8) | 101(34.02) |  | 149(49.7) | 151(50.3) |  |
| Education | | |  |  |  | **<0.001** |  |  | **<0.001** |
|  | None/Primary  (0-6 years) | | 10,746(44.6) | 9,328(81.8) | 2,081(18.2) |  | 10,057(88.1) | 1,361(11.9) |  |
|  | Secondary  (7-12+ years) | | 11,293(46.9) | 10,009(86.0) | 1,631(14.0) |  | 10,948(94.0) | 699(6.0) |  |
|  | NA | | 2,068(8.6) | 1,682(77.1) | 501(23.0) |  | 1,738(79.3) | 453(20.7) |  |
| Parity | | |  |  |  | **<0.001** |  |  | **<0.001** |
|  | 1^B^ | | 8,966(37.2) | 7,478(81.9) | 1,654(18.1) |  | 8,396(91.9) | 742(8.1) |  |
|  | 2 | | 5,534(23.0) | 4,987(86.0) | 812(14.0) |  | 5,371(92.6) | 432(7.4) |  |
|  | ≥3 | | 9,136(37.9) | 8,249(83.8) | 1,595(16.2) |  | 8,727(88.6) | 1,125(11.4) |  |
|  | Unknown | | 471(2.0) | 305(66.7) | 152(33.3) |  | 249(53.8) | 214(46.2) |  |
| C-section^C^ | | |  |  |  |  |  |  | **<0.001** |
|  | No | | 20,486(85.0) | - | - |  | 19,538(91.6) | 1,798(8.4) |  |
|  | Yes | | 3,590(14.9) | - | - |  | 3,178(81.7) | 711(18.3) |  |
|  | NA | | 31(0.1) | - | - |  | 27(87.1) | 4(12.9) |  |
| Vital status of  last born child | | |  |  |  | **<0.001** |  |  | **<0.001** |
|  | Liveborn | | 12,346(51.2) | 11,277(85.4) | 1,924(14.6) |  | 12,032(91.1) | 1,177(8.9) |  |
|  | Stillbirth | | 749(3.1) | 591(75.2) | 195(24.8) |  | 639(81.3) | 147(18.7) |  |
|  | Liveborn  -deceased later | | 1,448(6.0) | 1,271(83.1) | 258(16.9) |  | 1,345(87.8) | 187(12.2) |  |
|  | NA | | 9,564(39.7) | 7,880(81.1) | 1,836(18.9) |  | 8,727(89.7) | 1,002(10.3) |  |
| Antenatal visits^D^ | | |  |  |  | **<0.001** |  |  | **<0.001** |
|  | | No | 11,233(46.6) | 9,792(85.5) | 1,660(14.5) |  | 10,494(91.6) | 965(8.4) |  |
|  | | Yes | 11,122(46.1) | 9,457(83.6) | 1,860(16.4) |  | 10,065(88.8) | 1,263(11.2) |  |
|  | | NA | 1,752(7.3) | 1,770(71.9) | 693(28.1) |  | 2,184(88.5) | 285(11.5) |  |
| Antenatal treatment^E^ | | |  |  |  | 0.535 |  |  | 0.442 |
|  | Prophylaxis AZT | | 396(1.6) | 346(84.4) | 64(15.6) |  | 378(92.2) | 32(7.8) |  |
|  | cART | | 62(0.3) | 65(89.0) | 8(11.0) |  | 67(91.8) | 6(8.2) |  |
|  | No treatment* | | 108(0.4) | 81(84.4) | 15(15.6) |  | 88(91.7) | 8(8.3) |  |
|  | HIV negative | | 23,541(97.7) | 20,527(83.3) | 4,126(16.7) |  | 22,209(90.0) | 2,468(10.0) |  |
| Bissau resident | | |  |  |  | **<0.001** |  |  | **<0.001** |
|  | Yes | | 20,337(84.4) | 17,801(84.1) | 3,376(15.9) |  | 19,449(91.8) | 1,746(8.2) |  |
|  | No^F^ | | 2,053(8.5) | 1,715(75.6) | 553(24.4) |  | 1,693(74.5) | 580(25.5) |  |
|  | NA | | 1,717(7.1) | 1,503(84.1) | 284(15.9) |  | 1,601(89.5) | 187(10.5) |  |
| **Infant** | | |  |  |  |  |  |  |  |
| Low birth weight^G^ | | |  |  |  |  |  |  | **<0.001** |
|  | No | | 21,019(83.3) | - | - |  | 19,637(93.4) | 1,382(6.6) |  |
|  | Yes | | 4,213(16.7) | - | - |  | 3,096(73.5) | 1,117(26.5) |  |
| Twin birth | | |  |  |  | **<0.001** |  |  | **0.044** |
|  | | No | 24,106(94.5) | 20,468(85.7) | 3,426(14.3) |  | 21,556(90.1) | 2,358(9.9) |  |
|  | | Yes | 1,395(5.5) | 551(41.2) | 787(58.8) |  | 1,187(88.5) | 155(11.6) |  |

**Legend.** *p,* p-value; NA, Not available.

**^A^** Ethnic groups, each comprising less than 1% of the sample population.

**^B^** Separated, divorced or widowed.

^C^ Caesarean section.

^D^ Attended antenatal counselling and testing.

^E^ Received antenatal treatment for HIV positives in comparison with HIV negative. (lbw & stillbirth n=12 missing values)

^F^ Living in other regions of Guinea-Bissau (referral patients often due obstetric complications).

^G^ Excluding miscarriages (n=244) and missing values (n=25).

^H^ Including twin and triplet births.

^I^ Excluding miscarriages (n=244) and missing values (n=1).
