# Supplementary material for: HIV-1 and HIV-2 prevalence, risk factors and birth outcomes among pregnant women in Bissau, Guinea-Bissau: a retrospective cross-sectional hospital study
Source: Sci Rep. 2020 Jul 22;10:12174. doi: 10.1038/s41598-020-68806-5 (PMC7376101; doi:10.1038/s41598-020-68806-5)
Supplement: Supplementary file 2 — Supplementary Information 2. [file 41598_2020_68806_MOESM2_ESM.docx]

**HIV-1 and HIV-2 prevalence, risk factors and birth outcomes among pregnant women in Bissau, Guinea-Bissau: a retrospective cross-sectional hospital study**

**Authors:** Dlama Nggida Rasmussen^1,2,3*^ Noel Vieira^4^, Bo Langhoff Hønge^3,5,6^, David da Silva Té^7^, Sanne Jespersen^3,6^, Morten Bjerregaard-Andersen^3,8,9^, Ines Oliveira^3^, Alcino Furtado^3^, Magarida Alfredo Gomes^10^, Morten Sodemann^2,3^, Christian Wejse^3,6,11^, Holger Werner Unger^3,12,13^

**Affiliations:**

^1^ Department of Public Health, University of Southern Denmark, Odense, Denmark.

^2^ Department of Infectious Diseases, Odense University Hospital, Odense, Denmark.

^3^ Bandim Health Project, INDEPTH Network, Bissau, Guinea-Bissau.

^4^ Association Ceu e Terras, Bissau, Guinea-Bissau.

^5^ Department of Clinical Immunology, Aarhus University Hospital, Aarhus, Denmark.

^6^ Department of Infectious Diseases, Aarhus University Hospital, Aarhus, Denmark.

^7^ National HIV Programme, Secretariado Nacional de Luta Contra le Sida, Ministry of Health, Guinea-Bissau.

^8^ Department of Endocrinology, Hospital of South West Denmark, Esbjerg, Denmark.

^9^ Research Center for Vitamins and Vaccines, Statens Serum Institut, Copenhagen, Denmark.

^10^ Department of Obstetrics and Gynaecology, Simão Mendes National Hospital, Bissau, Guinea-Bissau.

^11^ GloHAU, Center for Global Health, Department of Public Health, Aarhus University, Aarhus, Denmark.

^12^ Centre for Maternal and Newborn Health, Liverpool School of Tropical Medicine, Liverpool, United Kingdom.

^13^ Department of Medicine at the Doherty Institute, The University of Melbourne, Australia.

**S1 Checklist.** STROBE Statement—checklist of items that should be included in reports of observational studies.

HIV-1 and HIV-2 prevalence, risk factors and birth outcomes among pregnant women in Bissau, Guinea-Bissau: a retrospective cross-sectional hospital study.

|  | Item No. | Recommendation | Page  No. | Relevant text from manuscript |
| --- | --- | --- | --- | --- |
| **Title and abstract** | 1 | (*a*) Indicate the study’s design with a commonly used term in the title or the abstract | 1 | “A retrospective cross-sectional study” |
|  |  | (*b*) Provide in the abstract an informative and balanced summary of what was done and what was found | 2 | “Abstract” |
| Introduction | | | |  |
| Background/rationale | 2 | Explain the scientific background and rationale for the investigation being reported | 3-4 | “Introduction” |
| Objectives | 3 | State specific objectives, including any prespecified hypotheses | 4 | “In this retrospective study, we assessed changes in HIV-1 and HIV-2 prevalence, factors associated with HIV infection, birth outcomes and the provision of ART as part of PMTCT services over a five-year period at the national maternity ward in Bissau, Guinea-Bissau..” |
| Methods | | | |  |
| Study design | 4 | Present key elements of study design early in the paper | 5 | “We conducted a retrospective cross-sectional survey exploring HIV prevalence, risk factors of HIV, treatment provision, and birth outcomes (low birth weight (LBW) and stillbirth) drawing on data routinely collected through the BHP surveillance system at HNSM from June 2008 until May 2013.…” |
| Setting | 5 | Describe the setting, locations, and relevant dates, including periods of recruitment, exposure, follow-up, and data collection | 4 & 5 | “The study was conducted by the Bandim Health Project (BHP) (http://www.bandim.org), at the Simão Mendes National Hospital (HNSM) maternity ward located in Bissau. The BHP, a health and demographic surveillance site present in Guinea-Bissau for four decades, routinely collects demographic and clinical data on all deliveries at the HSNM. This public facility is the principal provider of comprehensive obstetric care in Guinea-Bissau and approximately 90% of women who deliver at the facility are residents of the country’s capital, Bissau.,” &  “..from June 2008 until May 2013. “ |
| Participants | 6 | (*a*) *Cohort study*—Give the eligibility criteria, and the sources and methods of selection of participants. Describe methods of follow-up  *Case-control study*—Give the eligibility criteria, and the sources and methods of case ascertainment and control selection. Give the rationale for the choice of cases and controls  ***Cross-sectional study***—Give the eligibility criteria, and the sources and methods of selection of participants | 5 | “All women presenting to HNSM for delivery or immediate postpartum care who had been tested for HIV were included in this study.” |
|  |  | (*b*) *Cohort study*—For matched studies, give matching criteria and number of exposed and unexposed  *Case-control study*—For matched studies, give matching criteria and the number of controls per case | - | N/A |
| Variables | 7 | Clearly define all outcomes, exposures, predictors, potential confounders, and effect modifiers. Give diagnostic criteria, if applicable | - | N/A |
| Data sources/ measurement | 8* | For each variable of interest, give sources of data and details of methods of assessment (measurement). Describe comparability of assessment methods if there is more than one group | *-* | N/A |
| Bias | 9 | Describe any efforts to address potential sources of bias | 8 | “adjustment for birth outcomes was made for clustering of twins using a specific pair number” |
| Study size | 10 | Explain how the study size was arrived at | 5 | “All women presenting to HNSM for delivery or immediate postpartum care who had been tested for HIV were included in this study.” |

Continued on next page

| Quantitative variables | 11 | Explain how quantitative variables were handled in the analyses. If applicable, describe which groupings were chosen and why | 7 | “Outcome variables i.e. HIV status, low birth weight and stillbirths were dichotomized (0=absent, 1=present). Continuous explanatory variables were grouped categorically..” |
| --- | --- | --- | --- | --- |
| Statistical methods | 12 | (*a*) Describe all statistical methods, including those used to control for confounding | 7-8 | “Factors associated with HIV-serostatus and birth outcomes were determined using univariate and multivariate logistical regression models. The multivariate analysis was fitted with statistically significant covariates (p<0.05) from the univariate analysis as determined by the Wald’s test. Birth outcomes i.e. stillbirths and low birthweight (LBW) were examined according to HIV status adjusted for significant co-variates..” &  “Adjustments in the logistical regression models for birth outcomes were made for clustering of twins using af specific pair number. Missing values were included in the logistical regression models. Trends over time (calendar year) for HIV prevalence and treatment were determined using Pearson’s χ2 test. Due to lack of data to confirm self-reported antenatal treatment regimens and reasons for selecting a given treatment at labour we opted to assess antenatal and treatment at labour separately. A p-value of <0.05 was considered significant.  .” |
|  |  | (*b*) Describe any methods used to examine subgroups and interactions | - |  |
|  |  | (*c*) Explain how missing data were addressed | 8 | “Missing values were included in the logistical regression models.” |
|  |  | (*d*) *Cohort study*—If applicable, explain how loss to follow-up was addressed  *Case-control study*—If applicable, explain how matching of cases and controls was addressed  *Cross-sectional study*—If applicable, describe analytical methods taking account of sampling strategy |  |  |
|  |  | (*e*) Describe any sensitivity analyses |  |  |
| Results | | | | |
| Participants | 13* | (a) Report numbers of individuals at each stage of study—eg numbers potentially eligible, examined for eligibility, confirmed eligible, included in the study, completing follow-up, and analysed | 9 | “Participant characteristics” |
|  |  | (b) Give reasons for non-participation at each stage |  | NA |
|  |  | (c) Consider use of a flow diagram | Figure 1-STROBE |  |
| Descriptive data | 14* | (a) Give characteristics of study participants (eg demographic, clinical, social) and information on exposures and potential confounders | Table 1 |  |
|  |  | (b) Indicate number of participants with missing data for each variable of interest | 9 | “**Table 1.** Baseline characteristics of pregnant women. NA, Not available.” |
|  |  | (c) *Cohort study*—Summarise follow-up time (eg, average and total amount) | - | Not relevant |
| Outcome data | 15* | *Cohort study*—Report numbers of outcome events or summary measures over time | *-* | Not relevant |
|  |  | *Case-control study—*Report numbers in each exposure category, or summary measures of exposure | *-* | Not relevant |
|  |  | *Cross-sectional study—*Report numbers of outcome events or summary measures | *8* | Please see results. |
| Main results | 16 | (*a*) Give unadjusted estimates and, if applicable, confounder-adjusted estimates and their precision (eg, 95% confidence interval). Make clear which confounders were adjusted for and why they were included | Results | “Table 2,3 and 4. e.g. Adjusted for age by groups, ethnicity, marital status, education, parity, vital status of last born child, previous antenatal counselling, Bissau resident (resident or referral patient for another region) and twin birth.” |
|  |  | (*b*) Report category boundaries when continuous variables were categorized | - | N/A |
|  |  | (*c*) If relevant, consider translating estimates of relative risk into absolute risk for a meaningful time period | - | N/A |

Continued on next page

| Other analyses | 17 | Report other analyses done—eg analyses of subgroups and interactions, and sensitivity analyses | - | N/A |
| --- | --- | --- | --- | --- |
| Discussion | | | | |
| Key results | 18 | Summarise key results with reference to study objectives | 13-14 | “This retrospective cross-sectional study examined HIV-1 and HIV-2 prevalence as well as risk factors for HIV, PMTCT treatment coverage and birth outcomes at the largest maternity ward in Guinea-Bissau. It shows a significant decline in HIV-1, HIV-2, HIV-1/2 prevalence among pregnant women presenting for labour during a 5-year period....” |
| Limitations | 19 | Discuss limitations of the study, taking into account sources of potential bias or imprecision. Discuss both direction and magnitude of any potential bias | 16-17 | “…Our research has several limitations. First, the cross-sectional nature of this study means we are unable to determine trends in incidence and causality. One-third of women presenting for labour during the study period were not counselled or tested for HIV. These women were not included in this study due to missing information on HIV status presenting a potential selection bias...” |
| Interpretation | 20 | Give a cautious overall interpretation of results considering objectives, limitations, multiplicity of analyses, results from similar studies, and other relevant evidence | 12-17 | “Discussion” |
| Generalisability | 21 | Discuss the generalisability (external validity) of the study results | 16 | “HIV prevalence was determined based on women presenting at the HNSM maternity ward and may differ from women giving birth at other urban health centres or at home.” |
| Other information | |  | | |
| Funding | 22 | Give the source of funding and the role of the funders for the present study and, if applicable, for the original study on which the present article is based | 21 | “This study was made possible by a research grant from the Albert McKern Bequest, Edinburgh, UK to HWU, and financial support from the Department of Infectious Diseases, Odense University Hospital, and the Department of Public Health, Research Unit for General Practice, University of Southern Denmark, to DNR.” |

*Give information separately for cases and controls in case-control studies and, if applicable, for exposed and unexposed groups in cohort and cross-sectional studies.

**Note:** An Explanation and Elaboration article discusses each checklist item and gives methodological background and published examples of transparent reporting. The STROBE checklist is best used in conjunction with this article (freely available on the Web sites of PLoS Medicine at http://www.plosmedicine.org/, Annals of Internal Medicine at http://www.annals.org/, and Epidemiology at http://www.epidem.com/). Information on the STROBE Initiative is available at www.strobe-statement.org.
